# An Exercise Program Designed for Children with Attention Deficit/Hyperactivity Disorder for Use in School Physical Education: Feasibility and Utility

**DOI:** 10.3390/healthcare7030102

**Published:** 2019-09-04

**Authors:** Alyx Taylor, Dario Novo, David Foreman

**Affiliations:** 1 Department of Physiology, School of Psychology, Sport and Physical Activity, AECC University College, Bournemouth BH5 2DF, UK; 2 Independent Researcher, London SW11 5BW, UK; 3 Institute of Psychiatry, Psychology and Neuroscience, King’s College London, London SE5 8AF, UK

**Keywords:** symptom-reduction, ADHD, treatment adjunct, PE, physical activity

## Abstract

Moderate to high intensity exercise can improve cognitive function and behavior in children including those with attention-deficit/hyperactivity disorder (ADHD). However, exercise with long periods of the same activity, or inactivity can fail to engage or maintain their attention. This study examined the effect of exercise sessions developed to engage children with ADHD. Twelve children (10–11 years), six with a diagnosis of ADHD and six with no diagnosis, undertook 40-min sessions of short-duration, mixed activities bi-weekly for eleven weeks. ADHD symptoms and exercise enjoyment were recorded before six and eleven weeks of intervention. Teacher-reported data showed ADHD symptoms were significantly decreased in the children with ADHD, with a moderate to large effect size. There were no changes in the control group. All children indicated equal enjoyment of the exercise sessions. Specially designed exercise sessions stimulate and maintain engagement by children with ADHD and may reduce ADHD symptom levels in the school environment. The method that supports inclusive practice in physical education (PE) was successfully transferred to the study school and led by the usual class teacher. Children evaluated the exercises as acceptable and enjoyable for those with and without ADHD. This inclusive exercise method might help children manage ADHD symptoms.

## 1. Introduction

Attention-deficit/hyperactivity disorder (ADHD) is a syndrome of inattention, impulsivity, and over-activity in variable proportions, sufficient to cause impairment in functioning. Systematic review and meta-analysis data estimate the prevalence to be between 5 and 7% [1,2].

Children with ADHD may struggle to maintain age appropriate behavior and show poor social competence or emotional dysregulation [3,4,5]. This can present difficulties for children with ADHD in co-operative activities at school. They may also find themselves socially excluded by other pupils, which is distressing for the children and their families [6].

Recognizing that children with ADHD need a different approach to teaching and learning, DuPaul et al. [7], developed an innovative strategy of consistent monitoring in real situations in school, to engage and support children with their behavior and school-work. This involved intensive co-operative effort by both parents and teachers. Vygotsky’s zone of proximal development provides a theoretical framework to describe the situation the children were able to work in [8]. That is, the close guidance enabled them to function at a level beyond their current stage of development. Tasks were of short duration and children were given options that all led to the same learning outcomes. This strategy was successful with regular lessons in the classroom [7].

Studies involving children with ADHD have been undertaken to examine the effects of exercise at moderate to high levels of intensity under experimental conditions. Improvements in motor skills, cognitive function, and behavior have been found after acute and long-term exercise [9,10,11]. However, different frequencies of exercise have been used in the studies, from a single acute exercise session to multiple sessions per week over a period of months [12,13,14]. Due to this and other differences in methodology, the specific exercise and related benefits for children with ADHD remain unclear and further research has been proposed [12,13,14]. There is also evidence that exercise may have positive effects on both social and cognitive development in the general population of children [15]. Furthermore, it has been argued that ADHD could be an extreme level of traits that occur in a continuum in the general population [16]. Therefore, it might be predicted that an exercise intervention would have a varying effect, depending on the level of ADHD symptoms.

Interest in exercise as a possible adjunct to treatment for ADHD has been stimulated by concern over side effects from medication and difficulty implementing behavioral interventions [17]. However, the way in which exercise might affect the brain and behavior of those with ADHD is not yet fully understood. Differences have been observed in the brain structure and function of those with ADHD, including the prefrontal cortex and its connections to striatal, parietal, and cerebellar circuits [18]. Stimulant medication that increases dopamine, norepinephrine, or both, leading to increased activation of the PFC and its connections, has been associated with improved cognitive control in people with ADHD [19,20]. It is possible that exercise that stimulates the release of these hormones may be having a similar effect on the brain.

The evidence indicates that exercise might benefit those with ADHD more than those with few or no symptoms of ADHD. In addition, no studies reported adverse effects of exercise [13,16]. Therefore, it appears that physical education (PE) lessons for the whole class could be especially beneficial for children with ADHD. However, the standard PE lessons in school can be challenging for children with ADHD due to long periods of the same activity, or periods of inactivity in team sports. To overcome this, the strategy used by DuPaul et al. [7] might motivate the children with ADHD in PE lessons. That is, providing choice and multiple short-duration exercises in PE lessons might reduce ADHD symptoms.

It is also interesting that progressive teaching methods have addressed the need for children with ADHD to have some physical movement during seated classroom activities [21]. Children might be asked to undertake a small task for the teacher, such as collecting books, or they might be encouraged to squeeze a stress ball at their desk [22,23]. Further advice is that children with ADHD should not miss break-times or PE lessons. However, specific activities or adjustments to the PE lessons are not included in this advice.

**Figure A1:**
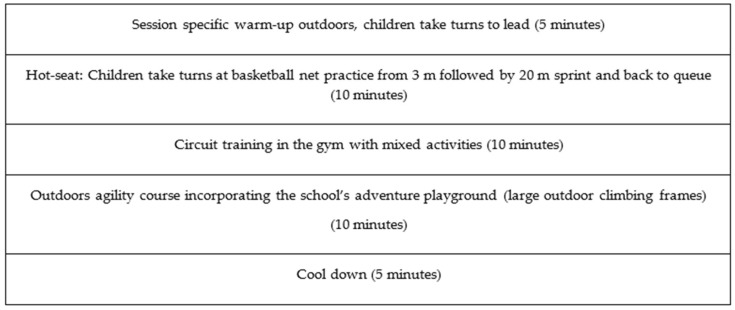
A moderate to high intensity exercise session.

Previously, this research group developed a series of 40-min, moderate to high intensity mixed-activity exercise sessions for children with ADHD, suitable for normal school facilities [24]. These include both indoor and outdoor activities, easily adaptable to the available facilities. An example exercise session is provided in Figure A1 and full details of the exercises are provided in the Supplementary Materials.

The exercise sessions were developed with children diagnosed with ADHD. However, these sessions were not tested by children who do not have ADHD. Current developments in education policy encourage inclusive strategies for children with special educational needs including ADHD [25]. Therefore, any new exercise sessions must be acceptable to all children in the class. The current feasibility study was designed to deliver the exercise sessions in the context of normal school activities for children with and without ADHD. The aim was to examine the acceptability and the impact on symptoms of exercise at school designed for children with ADHD.

## 2. Materials and Methods

### 2.1. Participants

This feasibility study followed a repeat-measures design to examine the effect of an exercise program developed for children with ADHD. An ADHD study group and an equally sized, age-matched control group were included. Ethical approval for the study was granted by the University of West London, FREC04. Children aged 10–11 years (school years five and six) attending a school in the UK were invited with their parents or guardians to take part. Exclusion criteria were the diagnosis of a cardiovascular condition, a respiratory problem, or a current infection [26]. Inclusion criteria for the ADHD study group were age 10–11 years and a diagnosis of ADHD. Inclusion criteria for the control group were age 10–11 years with no diagnosis of a mental health disorder. Twelve children who met the criteria joined the study: six in the ADHD study group and six age-matched class-mates in the control group. The study group consisted of five boys and one girl of whom four were white and two were Asian British. The control group consisted of three boys and three girls who were all white. All the children diagnosed with ADHD continued taking their normal medication. Informed consent was obtained from both the parents or guardians and the children who chose to take part.

### 2.2. Procedure

Afternoon exercise sessions were held twice weekly over one 12-week term. Baseline measures of ADHD symptoms for children in the study and control groups were recorded by both parents and teachers before the exercise intervention began. Activity sessions included different combinations of activities to stimulate interest. Each session started with a session-specific warm-up for 5–10 min with children taking turns to lead. This was followed by two different gym-based and outdoor blocks of mixed activity, each lasting 10 min with a mini-break (20–30 s) between. The session then finished with a 5–10-min cool-down. All activities had previously been chosen by a sports scientist and sports psychologist to be engaging for the children and to enable a moderate to intense work-rate. The researcher used existing facilities and equipment belonging to the school. In addition, the exercise sessions can be adapted to utilize the existing equipment of each school to avoid extra cost. The activities had been tested and confirmed as acceptable by children with ADHD [24]. The exercise sessions were mentally as well as physically demanding. They required the children to pay attention to the tasks and to the instructions from the leaders, to wait for their turn, and to work with other children in shared activities. This required focused attention, because the children did not all do the same activity at the same time. An example of this was two children taking a turn at a balancing challenge, while the others were doing shuttle runs.

Child-friendly hand signals were used during exercise sessions to monitor progress and enjoyment during each activity, but these signals are not a validated rating scale and the responses were not enumerated. The children’s own special learning assistants (SLAs) were available to assist should any child need to withdraw from a session. The total ADHD symptoms were recorded by the class teachers and parents at three time-points: baseline before the intervention started, then on weeks six and eleven of the exercise intervention.

### 2.3. Measures

The home and school versions of the ADHD Rating Scale-IV were used to measure the symptoms of ADHD [27]. This psychometric rating scale was originally developed by DuPaul [28]. The parent-rated (home) and teacher-rated (school) versions were shown to have high internal consistency, test–retest reliability, and criterion-related validity [29]. Both parent and teacher versions have 18 items describing symptoms of ADHD, developed from the Diagnostic and Statistical Manual of Mental Disorders-IV criteria for ADHD [27]. Each item is scored from 0–3 on a four-point Likert scale, with a score of 0 representing never or rarely and a score of 3 representing very often. An example of a hyperactivity–impulsivity item is “Leaves seat in classroom or other situations in which remaining seated is expected” and an example of an inattentive symptom item is “Does not follow through on instructions and fails to finish work” [28].

### 2.4. Statistical Analysis

The study group was derived from the limited number of children with ADHD in years 5 and 6 of one primary school. The small sample did not meet the criteria for normal distribution. Test–retest reliability was examined using Kendall’s tau due to the non-normal distribution, small sample size, and large number of tied ranks. The Wilcoxon signed ranks test was used to compare the ADHD scores for the study group and for the control group pre to post intervention. Univariate ANOVA, with Bonferroni correction, was then used to examine ADHD scores for the study group over the 12-week period of the study. Planned contrasts were used for within participant comparisons between time-points.

## 3. Results

Twelve children took part and completed the first six weeks of the study. The hand signals during the exercise sessions and the verbal responses from the control group children confirmed their enjoyment of the exercises matched that of the children with ADHD. Activities chosen as favorites by the children in the previous work were confirmed as favorites by all the children in the current study giving the thumbs up signal.

The test–retest reliability, of the teacher-rated ADHD symptoms scores, was examined by a Kendall’s tau calculated for teacher-rated symptoms scores at six weeks compared with baseline. There was a positive correlation between the two variables τ = 0.881, n = 12, *p* < 0.001, indicating good test–retest reliability.

**Figure 1:**
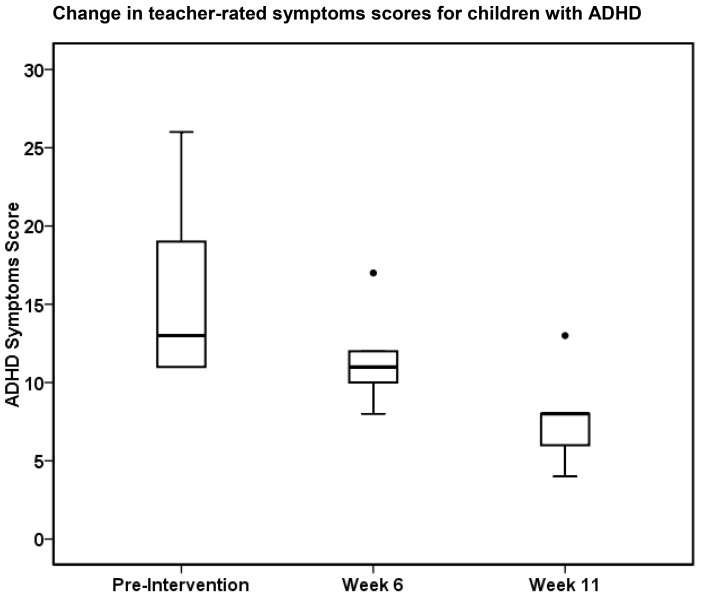
The graph shows a statistically significant reduction in teacher-rated symptom scores (median and range) for the children with ADHD (*n* = 6) after 11 weeks of exercise; *p* = 0.028.

The study group with ADHD completed all eleven exercise sessions (Table 1). The median (Mdn), teacher-rated ADHD symptom scores for the study group were significantly lower after eleven weeks of the physical exercise intervention (Mdn = 8.0) than before the intervention (Mdn = 13.0), z = −2.20, *p* = 0.028, r = −0.64 (Figure 1). The effect size was large, accounting for 40% of the variance. The parent-rated ADHD symptom scores for the study group followed the same trend as the teacher-reported data, but this did not reach statistical significance (Table 1).

All of the control group (*n* = 6) completed the first six weeks of the study. After week six, five of the control group were unable to complete all the exercise sessions. There were no differences in the teacher-rated or parent-rated symptom scores, which ranged from 0–2 at the three timepoints for the children in the control group.

A one-way repeated-measures analysis of variance (ANOVA) was conducted to evaluate the null hypothesis that there would be no change in the ADHD symptom level of the participants of the study group when measured before, during, and after the exercise sessions. Mauchly’s test showed the data met the assumption of sphericity. However, the Greenhouse–Geisser estimate of the departure from sphericity was *ε* = 0.74 and the correction was applied. There was a significant main effect of exercise on the symptom level reported by the teachers, *F*(1.49, 7.44) = 14.81, *p* = 0.004, *ω*^2^ = 0.34. The multivariate tests also showed that the ADHD symptom level was affected by the number of weeks of exercise undertaken. There was a significant effect of exercise on the ADHD symptom level reported by teachers, V = 0.82, *F*(2,4) = 9.33, *p* = 0.031. Planned contrasts were undertaken. The first contrast compared the teacher-rated ADHD scores at baseline with those after six weeks of the exercise sessions. This showed a significant reduction in ADHD scores from baseline to week 6 of exercise, *F*(1, 5), = 7.5, *p* = 0.041, with a large effect size r = 0.78. The second showed a significant reduction in teacher-rated ADHD scores from week six to week eleven of the exercise sessions, *F*(1, 5), = 14.76, *p* = 0.012, with a large effect size r = 0.86.

## 4. Discussion

In this study the use of exercise sessions designed for children with ADHD in PE lessons was associated with a significant reduction in ADHD symptoms observed by the teachers in school. In addition, the teachers informed the researchers that they had observed a general increase in engagement in learning activities in the classroom by the children with ADHD. The observations were spontaneous and qualitative, because no quantitative measure of engagement in sedentary classroom activities was included in this study. Together, the results and observations suggest these exercise sessions may be a practical way to reduce ADHD symptoms through regular school PE. The program applied in this study is distinguished from other exercise programs used in previous research by its original elements including multiple different activities with short periods of time spent on each. While this differs from other programs, the use of moderate to high intensity exercise is similar to previous work. In addition, the observed change in behavior associated with the exercise intervention supports previous findings [10,12,13,14]. Furthermore, this indicates that a randomized controlled trial might demonstrate cognitive improvement as well as improvement in behavior equivalent to previous results following physical exercise in the laboratory setting [30,31] and in extra-curricular activities [32].

Following the pattern of the teacher-rated ADHD symptom reduction, the median parent-rated symptom scores (Table 1) showed a steady reduction, but this trend did not reach statistical significance. Factors that might contribute to this are firstly the baseline scores were all lower than the teacher-rated scores, so the reductions were smaller. Secondly, there was an extreme outlier at the final time-point in the parent-rated scores as shown by the decreased median score despite the very high maximum score (Table 1). Teacher-rated scores were not affected. This appears to reflect a particular influence not associated with the school environment. A future full-scale study would enable analysis to control for this.

All the children in the study, both with and without ADHD, engaged fully with the physical exercise sessions. Their feedback through the regular monitoring by the exercise leaders indicated that their evaluation and enjoyment of the exercise sessions matched that of the children with ADHD who had contributed to the original selection of the activities. The exercise leaders in this study were not the same as the exercise leaders in the previous school study, indicating that this program can be successfully led by other appropriately qualified people, for example class teachers who lead PE lessons as well as subject specialist PE teachers. In addition, the school in the current study was not the same as the one in the development study, so none of the class teachers or children had previously been involved. This transferability suggests that it is the design and content of the exercise sessions that is important in engaging and maintaining the attention of the children.

Research indicates that many school practitioners including teachers, special education needs coordinators, and specialists in pupil referral units use a range of strategies aimed to be generally inclusive rather than specifically designed interventions for ADHD children alone [33]. This may be part of an inclusive pedagogical approach as compared to an additional needs approach to inclusion [34]. The key to the inclusive approach is that the teacher provides appropriate options all aimed to achieve the same learning outcome and allows all the children in the class to choose one. In this way, children choosing specially adapted options are not singled out and therefore not stigmatized. In line with this inclusive pedagogical approach, Petrie et al. [35] explored the application of full inclusion to PE lessons with a primary focus on physical special needs. Multiple options were provided for the children, so they could all practice the same skill, for example balancing, at their own physical level. Therefore, to support the current pedagogical philosophy of inclusion the new exercise sessions should be acceptable to all the children. This feasibility study has provided the first evidence that physical activity sessions specifically designed with the help of children with ADHD can be acceptable and enjoyable for the other children in the same class.

### Limitations and Future Directions

This feasibility study took place in only one primary school, following the development of the method in a separate single primary school. Therefore, there were a limited number of children of the correct age with ADHD available to take part. So, the study design could not include a randomized allocation of children with ADHD into two groups, one undertaking the intervention plus normal PE lessons and the other just the normal PE lessons. An unforeseen limitation arose when places for a different activity for children were provided after week six, which eventually attracted five children and their parents from the control group. Future research involving more than one primary school could address both limitations. In addition, to mitigate against a possible Hawthorne effect, a future study should be designed to enable all the children of the school classes to take part if they want to, so the physical activity is not perceived as a special privilege for some children.

The rating scale used in this study only measures symptoms of ADHD so any other changes in the control group would not be detected. In addition, the drop-out rate of the control group was high, due to an unforeseen school timetable change at half-term. However, the control group had reported equal enjoyment through the six weeks of their participation. Therefore, further research is warranted, first, to determine whether the effect on symptoms in children with ADHD can be replicated in a larger sample over a longer period; second, to discover whether other measurable benefits (e.g., general well-being) can be detected in a non-ADHD control group; and finally, to test whether the general acceptability of the exercise sessions would enable their use as an inclusive activity in regular PE lessons, rather than an ADHD-specific intervention.

## 5. Conclusions

The results from this feasibility study indicate that specifically designed exercise sessions that stimulate engagement by the children with ADHD may be useful for symptom reduction. This study has shown that the exercise sessions and methods can be transferred to other schools and led by the general class teachers. This study has also provided the first evidence that these physical activity sessions are acceptable and enjoyable for other children. Therefore, they are potentially useful in the PE curriculum. While these positive results suggest the sessions could be used to increase engagement by children with ADHD in regular school PE classes and reduce symptoms in the classroom, a randomized controlled trial involving children with ADHD in both a study and a control group should be undertaken to test this over the full school year.

## Figures and Tables

**Table 1:** The table shows the parent and teacher-rated Attention Deficit/Hyperactivity Disorder (ADHD), symptom scores for the children with ADHD (*n* = 6) at each timepoint.

	Pre-Intervention Median (Range)	Week 6 Median (Range)	Week 11 Median (Range)
Teacher-rated ADHD scores	13 (11–26)	11 (8–17)	8 (4–13) *
Parent-rated ADHD scores	9 (8–17)	4 (0–12)	3.5 (0–26)

Note: ADHD—Attention-deficit/hyperactivity disorder; * *p* = 0.028.

## Supplementary Materials

The following are available online at https://www.mdpi.com/2227-9032/7/3/102/s1, Figure S1: Details of Exercise Activities.
