# Supplementary material for: An Exercise Program Designed for Children with Attention Deficit/Hyperactivity Disorder for Use in School Physical Education: Feasibility and Utility
Source: Healthcare (Basel). 2019 Sep 4;7(3):102. doi: 10.3390/healthcare7030102 (PMC6787573; doi:10.3390/healthcare7030102)
Supplement: Supplementary file 1 [file healthcare-07-00102-s001.pdf]

Supplementary Materials

ID	Sex	Age	Year	ADHDControl	TDPbase	TDP6	TDP11	PDPbase	PDP6	PDP11
1	1	11	6	1	26	17	13	17	0	0
2	2	10	5	0	0	0		0		
3	1	11	6	1	12	10	8	8	5	0
4	1	10	5	1	11	12	6	10	1	2
5	1	10	5	1	14	10	4	8	3	5
6	1	10	5	0	1	2	1	1	0	0
7	2	10	5	0	0	0		0	0	
8	2	10	5	1	19	12	8	12	12	26
9	1	10	5	0	0	0		0		
10	1	10	5	1	11	8	8	8	11	6
11	1	11	6	0	0	1		0	2	
12	2	10	5	0	1	2		0	0	

Male 1 Female 2   ADHD 1 Control 2

Figure S1. Details of Exercise Activities.

Hot Seat

- Children form a single file line approximately 3 meters in front of a basketball net mounted on a wall
- The front person attempts to shoot the basketball in the net
- Once he/she has had an attempt he/she sprints to a point 20 meters away and attempts to get back in line before it is his/her turn again
- This is repeated continuously with each child keeping their own score of balls they have successfully got in the net: the challenge is to beat their own previous top score

Circuit training

- Exercise stations were set up to include gymnasium equipment such as ropes and ladders. These were:
  - Bench bunny hops
  - Star-jumps
  - Up and over ladder climbs
  - Sit-ups
  - Mat drags
  - Squat thrusts
  - Upper body rope pulls
  - Vault jumps
  - Each station lasted 40 seconds and they moved around the circuit clockwise without rest until all stations were completed by each individual

Outdoors Agility Course

- Children complete the school's mini adventure playground without allowing any of their body parts to touch the floor
- 50-meter sprint
- Crawl under a large fabric "parachute"

- 30-meter sprint
- 3x shuttle runs collecting a rubber ring at each point
- 50-meter sprint
- 10 sit-ups, 10 star-jumps
- 30-meter sprint
- 10 burpees, 10 alternate leg squat thrusts
- 50-meter sprint to finish line
